# Structural Insights into the Activation of the RhoA GTPase by the Lymphoid Blast Crisis (Lbc) Oncoprotein*

**DOI:** 10.1074/jbc.M114.561787

**Published:** 2013-07-03

**Authors:** Marc Lenoir, Masae Sugawara, Jaswant Kaur, Linda J. Ball, Michael Overduin

**Affiliations:** From the ‡ School of Cancer Sciences, University of Birmingham, Birmingham B15 2TT, United Kingdom,; § Structural Genomics Consortium, University of Oxford, Oxford OX3 7DQ, United Kingdom, and; ¶ The Leibniz Institute of Molecular Pharmacology, Campus Buch, 13125 Berlin, Germany

**Keywords:** GTPase, Guanine Nucleotide Exchange Factor (GEF), Nuclear Magnetic Resonance, Oncogene, Protein Structure, Protein-Protein Interaction, Ras Homolog Gene Family, Member A (RhoA), Rho GTPases, AKAP, Lbc

## Abstract

The small GTPase RhoA promotes deregulated signaling upon interaction with lymphoid blast crisis (Lbc), the oncogenic form of A-kinase anchoring protein 13 (AKAP13). The onco-Lbc protein is a hyperactive Rho-specific guanine nucleotide exchange factor (GEF), but its structural mechanism has not been reported despite its involvement in cardiac hypertrophy and cancer causation. The pleckstrin homology (PH) domain of Lbc is located at the C-terminal end of the protein and is shown here to specifically recognize activated RhoA rather than lipids. The isolated dbl homology (DH) domain can function as an independent activator with an enhanced activity. However, the DH domain normally does not act as a solitary Lbc interface with RhoA-GDP. Instead it is negatively controlled by the PH domain. In particular, the DH helical bundle is coupled to the structurally dependent PH domain through a helical linker, which reduces its activity. Together the two domains form a rigid scaffold in solution as evidenced by small angle x-ray scattering and ^1^H,^13^C,^15^N-based NMR spectroscopy. The two domains assume a “chair” shape with its back possessing independent GEF activity and the PH domain providing a broad seat for RhoA-GTP docking rather than membrane recognition. This provides structural and dynamical insights into how DH and PH domains work together in solution to support regulated RhoA activity. Mutational analysis supports the bifunctional PH domain mediation of DH-RhoA interactions and explains why the tandem domain is required for controlled GEF signaling.

## Teaser

**Background:** The Lbc oncoprotein stimulates deregulated GTPase activity in RhoA.

**Results:** Although the Lbc DH domain can independently activate GTP exchange by RhoA, its PH domain also presents surfaces for DH and activated RhoA interaction.

**Conclusion:** Multiple sites on both structural domains of the Lbc scaffold control RhoA.

**Significance:** New sites for mechanism-based design of modulators of Lbc action are revealed.

## Introduction

Signaling relays between specific kinases and GTPases are mediated by AKAP^2^ scaffolds. The family of AKAP-lymphoid blast crisis (Lbc) proteins provides a critical paradigm for the regulated scaffolds that control RhoA GTPases (1). They mediate pathways involving the mitogen-activated protein kinase (MAPK) cascade (2) as well as PKA, PKCη, and PKD (or PKCμ) (3, 4). Their physiological complexes utilize these kinases as well as phosphatases such as Shp2 (5) to regulate GEF activity through docking sites including those offered by the DH and PH domains. The DH-PH pair thus represents a master node of GEF control and must be understood in its multiple states to effectively manipulate their interplay.

**FIGURE 1.**
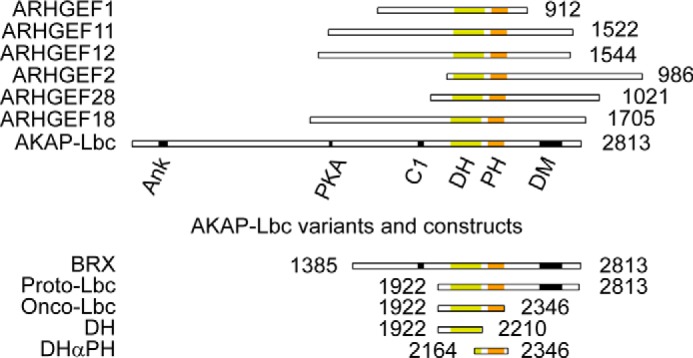
**Lbc RhoGEF family, AKAP13 variants, and constructs.** The orthologs and constructs of AKAP-Lbc are depicted with their constituent domains. The number of residues are indicated on the *right* for ARHGEF1 (also known as p115), ARHGEF11 (PRG or PDZRhoGEF), ARHGEF12 (LARG), ARHGEF2 (GEFH1), ARHGEF18 (p114), and ARHGEF28 (p190). The ankyrin binding site (*Ank*), PKA binding domain, C2, DH, PH, and dimerization (*DM*) domains are indicated. The DH and PH domains are indicated by *yellow* and *orange boxes*, respectively; other domains are represented by a *black box*.

Alternately spliced AKAP variants (see Fig. 1) were discovered in a screen for transforming genes from human myeloid leukemias. The isoforms include AKAP-Lbc, which is also known as AKAP13 (6) and Brx, which is specifically expressed in testis and estrogen-responsive reproductive tissues (7) and is linked to breast cancer (8) (Fig. 1). The regulated AKAP-Lbc scaffold is compromised in cases of chronic myeloid leukemia, breast cancer, and cardiac hypertrophy. A truncated form known as onco-Lbc was identified in patients suffering from myeloid leukemia (6). It is tumorigenic in mice and leads to oncogenic transformation of NIH 3T3 fibroblasts (9, 10). Relative to AKAP-Lbc, the oncogenic form, onco-Lbc, contains only the DH-PH tandem as well as a 70-residue N-terminal extension comprising residues 1922–2346 and induces constitutive GEF activity. Consequently it induces cell transformation in a Rho-dependent manner (11). Overexpression of AKAP-Lbc is found in uterine leiomyoma and may alter perception of mechanical stress (12). Cardiac hypertrophy and remodeling of the heart following stress also involve AKAP-Lbc signaling (13). Together these findings suggest that the Lbc family forms a critical trigger for mitogenic signaling, deregulation of which has dire consequences. This realization has stimulated growing interest focused on Lbc for drug discovery (14, 15). Moreover, as several of the ∼70 such DH-PH scaffolds in the human genome are oncogenic, additional related therapeutic targets may emerge (16).

The tandem DH-PH module is a prime target as it provides the core functionality required for RhoGEF activation. It captures the GDP-bound RhoA and stabilizes the nucleotide-free form until GTP is loaded and then released. Crystal structures of other DH-PH tandems indicate that the DH domain is structurally well conserved with variations in the length of its C-terminal helix and its orientation with the PH domain influencing their specific effects on GTPases (17). However, the specific relationships between AKAP-Lbc domains and their partners including RhoA, actin filaments (12), Gα proteins (4), and the plasma membrane lipids (18) remain unclear.

The interactions mediated by DH-PH scaffolds provide complex opportunities to regulate GTPase activity. Multiple positive and negative feedback loops can be mediated by the PH domain (19), a linker region at the N terminus of the DH domain, phosphorylation, lipids, and dimerization motifs. Activation results from removal of the C terminus of AKAP-Lbc (10). A leucine zipper found here mediates oligomerization and autoinhibition (20). Recently it was shown that the PH domains of Lbc family RhoGEFs bind to membrane-tethered RhoA-GTP and promote positive feedback (21). However, the exact Lbc mechanism remains unknown with no structures of any family member having been published.

Most interesting are the unique ligand interactions of Lbc DH-PH scaffolds that could account for their specific activities (22). Defining the structural basis of such interactions is necessary for designing selective molecular probes and inhibitors. Here we present solution structures of onco-Lbc and characterize the interactions among its DH and PH domains, RhoA, and lipids. By mapping and mutating the key residues, the mechanisms by which DH and PH domains communicate and integrate signals to control GTPase activity are revealed.

## EXPERIMENTAL PROCEDURES

### 

#### 

##### Protein Purification

The cDNA of human AKAP13 (Harvard database identification number HsCD00399180) corresponding to onco-Lbc (residues 1922–2346) or the isolated DH domain (1992–2210) was subcloned into a pGEX-6P-1 vector (GE Healthcare) between BamHI/SalI restriction sites and expressed in *Escherichia coli* BL21(DE3) cells. The production of the AKAP-Lbc construct encompassing residues 2164–2346 (“DHαPH”) was as described previously (23). Expression was induced overnight by addition of 1 mm isopropyl 1-thio-β-d-galactopyranoside at 18 °C. The cells were resuspended in phosphate-buffered saline buffer, pH 7.3 and 0.5 mm tris(2-carboxyethyl)phosphine and lysed, and soluble protein was purified on GST columns (GE Healthcare). Subsequently, the GST tag was cleaved with PreScission protease (GE Healthcare). Onco-Lbc constructs were further purified by size exclusion chromatography on an S75 26/60 Sephadex column using 50 mm Tris, pH 7.5, 150 mm NaCl, and 0.5 mm tris(2-carboxyethyl)phosphine. The identity and purity were assessed by SDS-PAGE. Mutations were generated using QuikChange mutagenesis kits (Stratagene), and the DNA sequences were verified by sequencing. Soluble RhoA (residues 1–181) was expressed overnight in *E. coli* BL21(DE3) at 18 °C and resuspended in 50 mm Tris, pH 8, 150 mm NaCl, 10 mm imidazole, 10% glycerol, 10 mm β-mercaptoethanol, 5 mm MgCl_2_, 100 μm GDP, and 0.1% Nonidet P-40. The protein was bound to a nickel column and eluted against an imidazole gradient. The fractions containing RhoA were pooled and further purified by size exclusion chromatography against a buffer containing 20 mm HEPES, pH 7, 100 mm NaCl, 5 mm MgCl_2_, and 2 mm tris(2-carboxyethyl)phosphine. RhoA-GTP and RhoA-GDP were prepared in buffers containing an excess (10×) of GTP or GDP in 20 mm Tris buffer, pH 8, 100 mm NaCl, 1 mm DTT (TB), and 10 mm EDTA. The excess nucleotide and EDTA were removed by exchange with TB containing 10 mm MgCl_2_.

##### NMR Spectroscopy

Uniformly labeled protein samples were prepared in M9 medium supplemented by ^15^NH_4_Cl or ^15^NH_4_Cl/[^13^C_6_]glucose as the sole source of nitrogen or carbon. The structure of the DHαPH domain (500 μm) of onco-Lbc was determined using NMR spectra acquired at 297 K on Varian Inova 800- and 900-MHz spectrometers equipped with triple resonance cold probes with enhanced ^13^C and ^1^H sensitivity and *z* axis gradients using assigned ^1^H, ^13^C, and ^15^N resonances (23). The protein samples were dissolved in H_2_O or 10% D_2_O and used for the acquisition of ^13^C- and ^15^N-resolved NOESY-HSQC experiments to estimate interproton distances from cross-peak volumes based on mixing times of 100 ms. The dihedral angles were derived from DANGLE (24), and hydrogen bonds were identified by deuterium exchange.

To monitor possible interactions with plasma membrane lipids by NMR, soluble lipid titrations were carried out using dihexanoyl derivatives of phosphatidylserine, PtdIns(4,5)P_2_, or PtdIns(3,4,5)P_3_ (Cayman Chemicals, Ann Arbor, MI) dissolved in the NMR sample buffer. Interactions with micelles were tested using dodecylphosphocholine with and without CHAPS (Sigma-Aldrich), which was added to help stabilize the protein.

##### NMR Structure Determination

The solution structures of the DHαPH domain were calculated with ARIA2.2 (25). A total of 100 structures were generated at each of the eight iterations in vacuum using torsion angle dynamics. The final refinement step was performed in explicit water. Twenty representative structures were selected based on their favorable energies and minimal violations as analyzed by PROCHECK (26). The backbone order parameters (*S*^2^) were computed using the RCI server (27).

##### Interaction between DHαPH and RhoA

The ^15^N-labeled DHαPH and RhoA-GDP samples were dialyzed against 20 mm Tris buffer, pH 7, 100 mm NaCl, 1 mm DTT, and 10 mm MgCl_2_. A series of ^15^N-resolved two-dimensional spectra were acquired in a solution containing DHαPH (100 μm) and after sequential addition of GTP (1 mm), RhoA-GDP (150 μm), onco-Lbc (4 nm), and finally 10 μl of calf intestinal alkaline phosphatase (Invitrogen) to cleave off nucleotide phosphate and thus to demonstrate the reversibility of the interaction.

##### Modeling

A structural model of onco-Lbc was built by Modeler using the DHαPH solution structure and structurally comparable DH domains from ARHGEF1 (p115), ARHGEF11 (PDZRhoGEF or PRG), ARHGEF12 (LARG), and Intersectin structures (Protein Data Bank codes 1TDX, 3ODO, 1XCG, and 1KI1). The orientations of onco-Lbc DH and PH domain residues were based on conserved DHαPH fold features common to the crystal structures and by the small angle x-ray scattering (SAXS) envelope. The Membrane Optimum Docking Area (MODA) and PIER programs (28, 29) were used as experimentally trained algorithms to predict direct membrane and protein binding surfaces, respectively, on the protein structures.

##### SAXS

Data were acquired at the X33 beamline at the European Molecular Biology Laboratory Hamburg outstation as described (30). Scattering patterns were collected at room temperature at protein concentrations between 2.0 and 6.1 mg/ml in 150 mm NaCl and 50 mm Tris buffer, pH 7.5. Background scattering caused by buffer alone was automatically subtracted from the protein scattering profiles. The data were processed using the ATSAS package (31). Radii of gyration (*R_g_*) and maximum particle sizes (*D*_max_) were determined using PRIMUS (32). DAMMIF (33) and DAMAVER (34) were used to generate the molecular envelope and average shape.

##### Guanine Exchange Experiments

Nucleotide exchange upon addition of onco-Lbc was measured on an LS55 PerkinElmer Life Sciences fluorescence spectrophotometer at 25 °C in TB containing 10 mm MgCl_2_. Nucleotide exchange activities used to compare the activities of AKAP-Lbc constructs in various conditions were carried out using 2 μm RhoA-GDP and 400 nm Mant-GTP (Invitrogen). For production of liposomes, a lipid stock of palmitoyloleylphosphatidylcholine (Avanti) at 2 mm was prepared in TB with 10 mm MgCl_2_ by successive freezing and thawing cycles. The resulting suspension was extruded through a 30-nm polycarbonate filter before the experiment. Exchange rates were measured from solutions containing RhoA loaded with Mant-GDP (500 nm) (Invitrogen) and the GTP analog GMP-PNP (100 μm) (Sigma). The rates of exchange were determined from the fluorescence change (excitation, 356 nm; emission, 440 nm) fitted to a single exponential. The GEF activities were calculated for concentrations of onco-Lbc ranging from 25 to 800 nm where the exchange activity varies linearly with the enzyme concentration.

##### Analytical Ultracentrifugation

The oligomeric state of AKAP-Lbc was assessed by sedimentation velocity experiments in a Beckman XLI ultracentrifuge using an eight-cell 50Ti rotor in 20 mm Tris, pH 7, 100 mm NaCl, 1 mm DTT, and 5 mm EDTA at 20 °C and 40,000 rpm. Proteins were detected from their absorbance at 280 nm. The viscosity and density of the solution were calculated from Sednterp (35), and the sedimentation coefficient distribution was calculated with Sedfit (36) using a continuous distribution model.

##### Surface Plasmon Resonance

A hexahistidine-tagged RhoA sample was exchanged overnight with nonhydrolyzable derivative GDPβS or GTPγS as described above. RhoA (200 nm; 30 μl) was coated on a nitrilotriacetic acid sensor chip on a Biacore 3000 instrument (GE Healthcare) at a flow rate of 10 μl·min^−1^ and rinsed with a pulse of imidazole (3 mm). The reference lanes were coated with hexahistidine-tagged ubiquitin. Experiments were carried out using a phosphate-buffered saline solution at pH 7.4 containing 1 mm MgCl_2_. Untagged onco-Lbc and DHαPH were injected (75 μl; 200-s dissociation time) in separate experiments to avoid cross-contamination between the RhoA-GDP and RhoA-GTP. Data were analyzed using BIAevaluation software.

## RESULTS

### 

#### 

##### Structure of AKAP-Lbc DHαPH Domain

**FIGURE 2.**
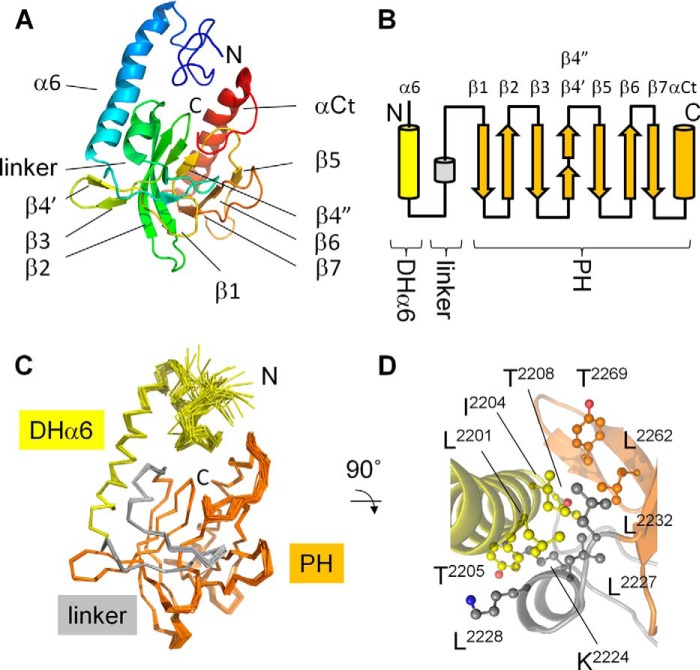
**Solution structure of the AKAP13 PH domain and DH α6 helix.** *A*, solution structure of AKAP13 PH domain and the C-terminal helix of the DH domain (DHαPH). The structure is colored from its N terminus (*blue*) to C terminus (*red*). The secondary structure elements and termini are labeled. *B*, the topology of the DHαPH fold includes the α6 helix of DH domain (*yellow*) followed by the linker region (*gray*) and the PH domain (*orange*). Secondary structures are labeled above with a bulge separating β4 into two ministrands, β4′ and β4″. *C*, the representative solution structures of DHαPH are superimposed, and the component domains are color-coded *yellow*, *gray*, and *orange* for the DHα6 helix, linker, and PH domain, respectively. *D*, the interface between the DH, PH, and linker is shown with side chains of residues involved in long range contacts represented with *sticks* and *balls*. The unambiguous distance restraints that link the DH and PH elements involve labeled residue pairs Leu^2201^-Leu^2232^, Leu^2201^-Leu^2227^, Ile^2204^-Leu^2227^, Tyr^2205^-Leu^2227^, Tyr^2205^-Lys^2224^, Tyr^2205^-Lys^2228^, Thr^2208^-Tyr^2269^, Thr^2208^-Leu^2262^, Thr^2208^-Lys^2224^, and Thr^2208^-Tyr^2269^. The solution structure was deposited under the Protein Data Bank code 2LG1.

To elucidate the respective orientation of the DH and PH domains in solution, we first determined the NMR structure of the PH domain and attached α6 helix of the DH domain. Constructs spanning only the canonical PH domain were markedly different in their NMR spectra and were also intrinsically unstable, suggesting that the α6 helix stabilizes the structure of the PH domain. This was despite extensive buffer screening of multiple AKAP-Lbc constructs using thermal shift assays with over 96 distinct buffer, salt, pH, and osmolyte conditions. This optimization did yield a stable construct in a physiological buffer suitable for NMR studies (50 mm phosphate buffer, pH 7.0, 150 mm NaCl, and 0.02% NaN_3_). The solution structure was calculated using 3564 distance, 234 dihedral angle, and 27 hydrogen bond restraints. The resulting ensemble of structures exhibited a backbone root mean square deviation of 0.34 Å for the structured elements between residues Gly^2186^ and Glu^2346^ (Fig. 2*A* and Table 1), whereas residues Ser^2162^–Ile^2185^ were unstructured. Thus, the minimal structural unit that is stably folded spans residues Gly^2186^–Glu^2346^. This represents what we term the DHαPH fold in recognition of the obligate integration of the PH fold with the last helix of the DH domain.

**TABLE 1:** **Structural statistics for the solution structures of the onco-Lbc DHαPH domain** r.m.s., root mean square; vdw, van der Waals; dihe, dihedral; cdih, *constrained dihedral.*

**Distance and dihedral constraints**	
Distance constraints	
^1^H-^1^H NOE	3537
Intraresidue (*i* = *j*)	1323
Small (|*i* − *j*| = 1)	517
Medium (2 ≥ |*i* − *j*| < 5)	335
Long range (|*i* − *j*| ≥ 5)	842
Ambiguous	520
Hydrogen bonds	27
Total dihedral angle restraints	
φ, Ψ	235

**Structure statistics**	
Violations*^a^*	
Distance constraints (Å) (>0.5 Å)	1.4
Dihedral angle constraints (°)	0.4
Deviations from idealized geometry	
Bond lengths (Å)	0.00674 ± 0.00038
Bond angles (°)	0.839 ± 0.027
Improper angles (°)	2.276 ± 0.364
Average pairwise r.m.s. deviation*^b^* (Å)	
Heavy, backbone	0.36, 0.76

**Energies (kcal·mol^−1^)**	
*E*_NOE_	756.1 ± 61.9
*E*_cdih_	8.6 ± 3.0
*E*_bond_	137.2 ± 14.5
*E*_improper_	279.4 ± 45.0
*E*_angle_	587.2 ± 37.8
*E*_vdw_	−10.6 ± 124.7
*E*_dihe_	1123.8 ± 25.9

**Ramachandran statistics (%)*^b^*^,^*^c^***	
Residues in core regions	76.3
Residues in allowed regions	21.6
Residues in generous regions	1.7
Residues in disallowed regions	0.4

*^a^* Averaged per structure.

*^b^* Residues Ile^2185^–Glu^2346^.

*^c^* Statistics were calculated from the 20 lowest energy structures out of 100 calculated.

**FIGURE 3.**
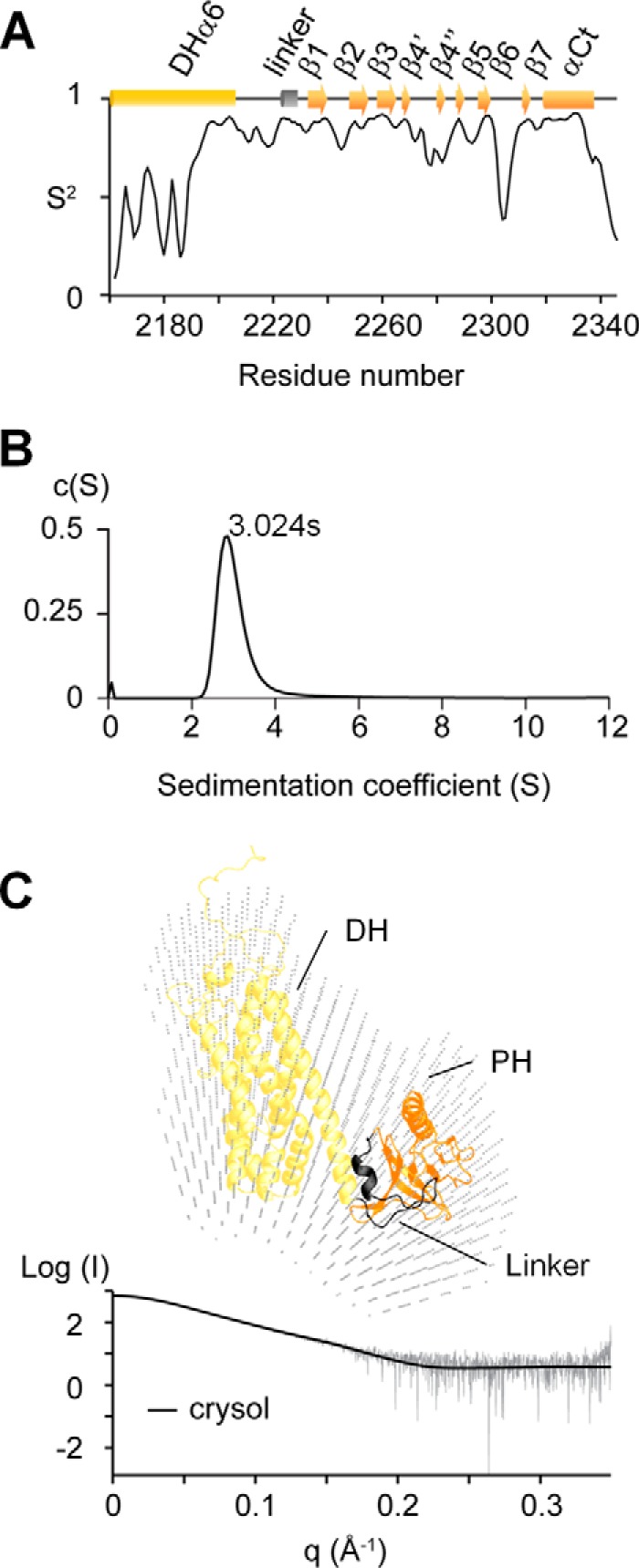
**Solution structure of the full-length onco-Lbc.** *A*, the dynamics of DHαPH is illustrated by the order parameters (*S*^2^) calculated using the RCI server (27). *B*, monomeric solution state of onco-Lbc as determined by velocity sedimentation. The distribution of the sedimentation coefficients is centered on 3.024 S, showing that onco-Lbc is monodispersed and monomeric in solution. *C*, interatomic distance distribution function for onco-Lbc calculated with PRIMUS. Models were generated with Modeler, and their theoretical scattering intensity was calculated with CRYSOL and fitted to the experimental data. The best fit calculated by CRYSOL between the experimental data and the model is represented in the *left panel* (χ^2^, 1.352). The best fit model of onco-Lbc is positioned in the molecular envelope generated with DAMMIF from the scattering pattern. Domains of onco-Lbc are color-coded as in Fig. 1.

The structure of the DHαPH domain of AKAP-Lbc differs in several significant ways from the canonical PH folds. A segment spanning eight amino acids (Phe^2271^–Thr^2279^) splits the β4 strand into two short strands, β4′ and β4″ and forms a bulge that obstructs the canonical lipid binding site found in PH domains (Fig. 2*B*). This element is structured based on NOE cross-peaks within the bulge (Leu^2274^-Lys^2277^ and Leu^2274^-Thr^2279^) and within the β4″ strand (Lys^2277^-Val^2280^ and Ser^2278^-Val^2280^) and the order parameters (27) (Fig. 3*A*), which indicate that this motif is structured. This represents a significant divergence from ARHGEF1, ARHGEF11, and ARHGEF12, which all possess an additional 11 residues here and form a highly flexible motif, suggesting a functional difference. The linker region between the DH and PH domains forms a short helix encompassing Lys^2224^–Arg^2229^ and an unstructured loop that folds back onto the strands of the PH domain (Fig. 2*C*). The linker helix interacts with the α6 helix through residues Leu^2201^, Ile^2204^, and Tyr^2205^ to form an ordered hydrophobic core that involves PH residues β3-Leu^2262^ and β4-Tyr^2269^ as well as linker residues Leu^2227^ and Leu^2232^ (Fig. 2*D*). This infers that Lbc-type PH domains only assume independently folded stable structures in solution when interdigitating their cores with the α6 and linker helices. Thus, β sheets of these PH domains may have evolved to endow unique functional and stabilizing features.

The dynamics of the DHαPH protein residues were characterized using secondary chemical shifts of backbone atoms (27). The order parameters calculated for individual structural elements within either DH or PH segments were very similar, indicating a single structure with significant dynamics concentrated in terminal residues before and after residues 2193 and 2340, respectively (Fig. 3*A*). This infers that the final four turns of the α6 helix are sufficient to form a structural unit that is as rigid as the attached PH domain. Together they form the structurally intact DHαPH fold. Only one loop exhibits significantly elevated dynamics, indicating a particularly rigid β sandwich fold. As such, the singularly flexible β6-β7 loop and its exposed residues including Met^2303^, Asp^2307^, and Met^2310^ may offer unique opportunities for induced binding of ligands as described below.

##### Modular Architecture of Onco-Lbc

Multimerization is an established means of RhoGEF control, and although some DH-PH tandems form monomers, dimer structures of others have been crystallized (Protein Data Bank codes 1X86, 1XCG, 3ODO, and 3KZ1). The oligomeric state of onco-Lbc remains indeterminate and hence was studied by analytical ultracentrifugation using sedimentation velocity experiments. The sedimentation coefficient of onco-Lbc was distributed around a single value (3.024 S), which demonstrated that onco-Lbc was monodispersed in solution (Fig. 3*B*). The corresponding estimated molecular mass of 54.8 kDa was consistent with a theoretical monomer size of 61.5 kDa.

The solution state formed by onco-Lbc was characterized by integrating the molecular envelope determined by SAXS and the structural model of the DH-PH tandem (Fig. 3*C* and Table 2). The SAXS envelope accommodated the structured DH and PH domains as well as the N terminus, which folded back onto the DH domain. The 49 residues at the extreme N terminus (Asn^1922^–Leu^1971^) of onco-Lbc are predicted to be disordered and could not be precisely modeled because of a lack of sufficiently similar three-dimensional structures. A series of 50 models were built, and their calculated scattering intensities were compared with the experimental data using CRYSOL (37) (Fig. 3*C*). The best matching model was fitted into the SAXS envelope and displayed the characteristic “chaise longue” shape of RhoGEF DH-PH domains (Fig. 3*C*). This suggests that the PH domain of onco-Lbc and its canonical lipid binding site and dynamic β6-β7 loop are positioned away from the active site of the DH domain that is formed by the conserved regions CR1 and CR3 and the α6 helix of the DH domain (16). These relative domain positions also infer that the DH and PH modules of onco-Lbc do not both simultaneously and directly control a GTPase molecule but rather that the PH domain could exert an indirect or separable role.

**TABLE 2:** **Structural parameter of onco-Lbc derived from SAXS data** *R_g_* and *D*_max_ are the radius of gyration and the maximum size, respectively. χ_shape_ and χ_model_ are the discrepancies between the calculated and experimental scattering curves for the molecular shape and the atomic model obtained by homology modeling, respectively.

*R_g_*	*D*_max_	χ_shape_	χ_model_
*nm*	*nm*		
2.97 ± 0.01	9.9 ± 0.5	1.015	1.352

##### The Guanine Exchange Activity Is Devolved to the DH Domain of AKAP-Lbc

**FIGURE 4.**
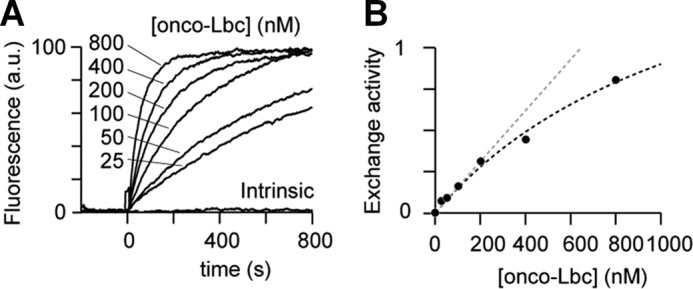
**RhoA nucleotide exchange as a function of onco-Lbc concentration.** *A*, the formation of RhoA-Mant-GTP was followed by fluorescence (excitation, 356 nm; emission, 440 nm) for onco-Lbc concentrations ranging from 0 and 800 nm. The AKAP protein was injected at time 0. *B*, the exchange activity of RhoA deviates from a straight line (*dotted gray line*) with increasing onco-Lbc concentrations and follows a hyperbolic function (*dotted black line*) indicative of a two-step mechanism. *a.u.*, arbitrary units.

**FIGURE 5.**
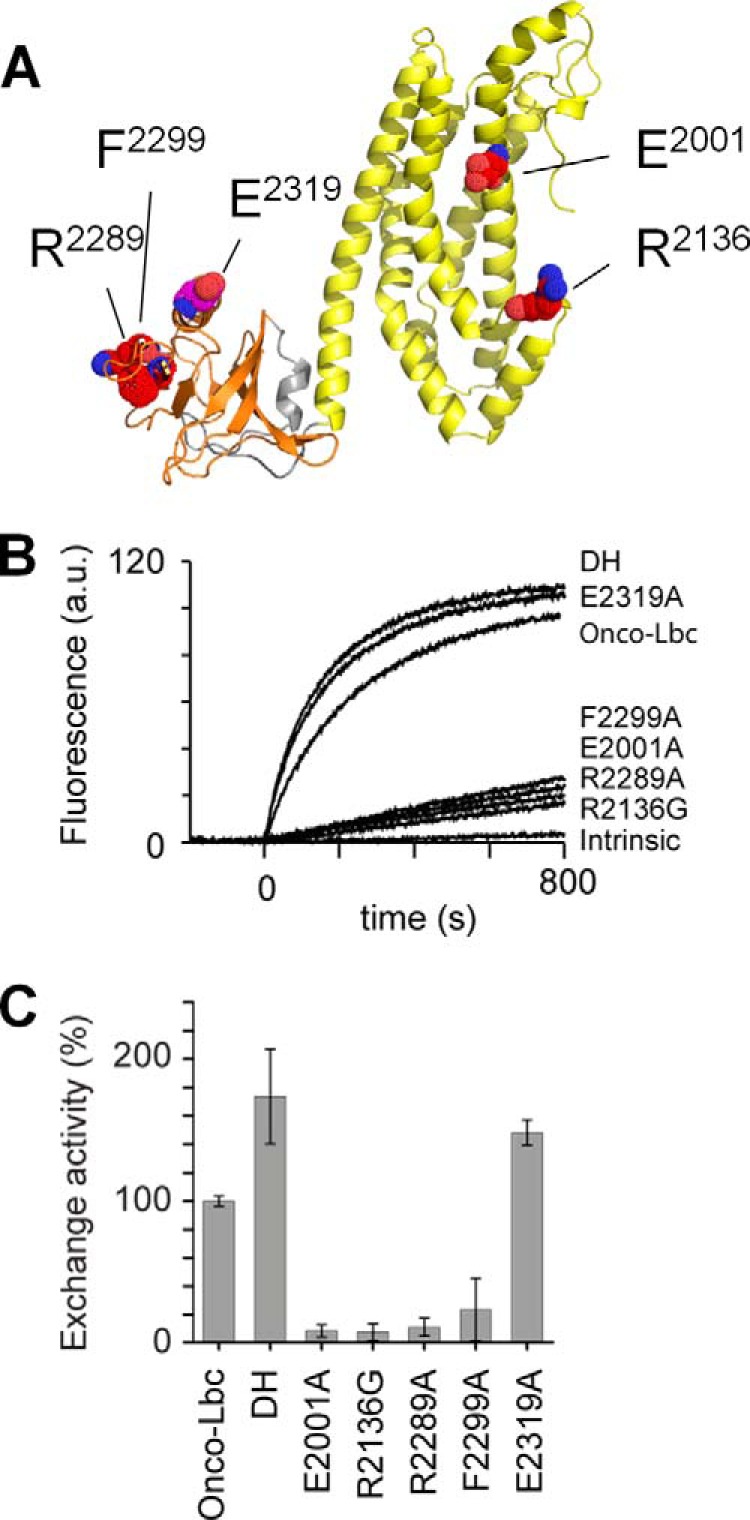
**GEF activity of onco-Lbc mutants.** *A*, the residues mutated in the DH-PH tandem are represented by atomic *spheres*. Mutations are colored according to the effects on GEF activity: *red* for inactivating except for Glu^2319^ (*magenta*), which is activating. *B*, the exchange activity of onco-Lbc mutants is compared with the wild-type onco-Lbc. The curves represent the exchange of GDP to Mant-GTP after injection of 200 nm onco-Lbc at time 0. Curves are labeled for each mutant. *C*, the exchange activities of wild-type onco-Lbc and mutants as calculated for GDP to Mant-GTP exchanges are depicted: onco-Lbc, 100 ± 3.6; DH, 173.6 ± 33.4; E2001A, 8.4 ± 4.4; R2136G, 7.4 ± 6.0; R2289A, 10.9 ± 6.5; F2299A, 23.3 ± 22.1; and E2319A, 148.0 ± 8.9. *a.u.*, arbitrary units. *Error bars* represent S.D.

To establish the GEF activity determinants, we first measured onco-Lbc effects over a concentration range (Fig. 4*A*). The activity varied in a hyperbolic manner over the range of concentrations used (Fig. 4*B*). This was consistent with other GEFs carrying a DH-PH tandem that catalyzes the GTP exchange in a two-step binding model (38). Next, to investigate the contribution of the PH domain, we compared the activities of onco-Lbc and its isolated DH domain (Fig. 5). This revealed that the Lbc DH domain is primarily responsible for mediating the GEF activity.

In other RhoGEFs related to Lbc, truncations of the PH domain have been associated with a significant loss of GEF activity (39, 40). Instead, in onco-Lbc, the deletion of the PH domain resulted in enhancement of GEF activity by a factor of 1.74 (Fig. 5). Conversely, the PH domain alone did not show GEF activity toward RhoA (data not shown) nor did its presence inhibit the reaction. The fact that the Lbc PH domain appears to be functionally dispensable can be explained by its unusual structural orientation whereby the αCt helix does not directly bind DH-bound RhoA unlike in ARHGEF11. Together the GEF results with the isolated DH and onco-Lbc constructs indicate that the Lbc PH domain exerts a unique inhibitory effect on the catalytic activation by the DH domain.

Lipid interactions were investigated as many PH domains including that of AKAP-Lbc associate with membranes (18, 41), and a homologous hydrophobic part of ARHGEF12 may contact lipids (39). In the case of onco-Lbc, its PH domain did not associate with phosphoinositides or phosphatidylserine derivatives. That is, there was an absence of NMR signal perturbations after these soluble ligands were titrated in. Moreover the addition of small unilamellar vesicles composed of palmitoyloleylphosphatidylcholine did not modify the nucleotide exchange activity detected by fluorescence (data not shown). This membrane-independent Lbc activity is consistent with the absence of exposed hydrophobic residues in the β1-β2 loop that usually mediate membrane insertion as well as the lack of a canonical phosphoinositide recognition motif.

For functional comparison, the specific exchange rates were contrasted between the onco-Lbc constructs and its orthologs (Table 3). The specific activity of onco-Lbc (3.92 × 10^3^ m^−1^ s^−1^) was an order of magnitude lower than that of ARHGEF12 (39), which had been acquired under similar conditions, whereas the isolated DH domain was only 4 times slower than that of ARHGEF12 (9.06 × 10^3^ m^−1^ s^−1^). Thus, activities of onco-Lbc and its DH domain are consistently lower than those of ARHGEF12. Its enhanced GEF activity when the PH domain is removed is in contrast to other Lbc-type RhoGEFs that display significant decreases of activity when the PH domain is truncated.

**TABLE 3:** **Specific exchange activities of onco-Lbc mutants**

Onco-Lbc	Specific exchange activity*^a^*	Relative activity
	*(s*^−*1*^ *m*^−*1*^*)* × *10^3^*	
DH-PH	3.92	1
DH	14.33	3.70
E2001A	1.07	0.27
R2136G	0.23	0.06
R2289A	0.68	0.17
F2299A	0.44	0.11
E2319A	7.30	1.86

*^a^* The specific exchange activity was calculated by fitting the decrease of fluorescence that accompanies the replacement of Mant-GDP by GMP-PNP to a single exponential function assuming a pseudo-first order rate of the reaction (*k*_obs_) and corrected by the intrinsic exchange activity of RhoA (*k*_intrinsic_) according to *k*_obs_/[onco-Lbc] − *k*_intrinsic_.

##### Mapping Activated RhoA Docking Site in Lbc

**FIGURE 6.**
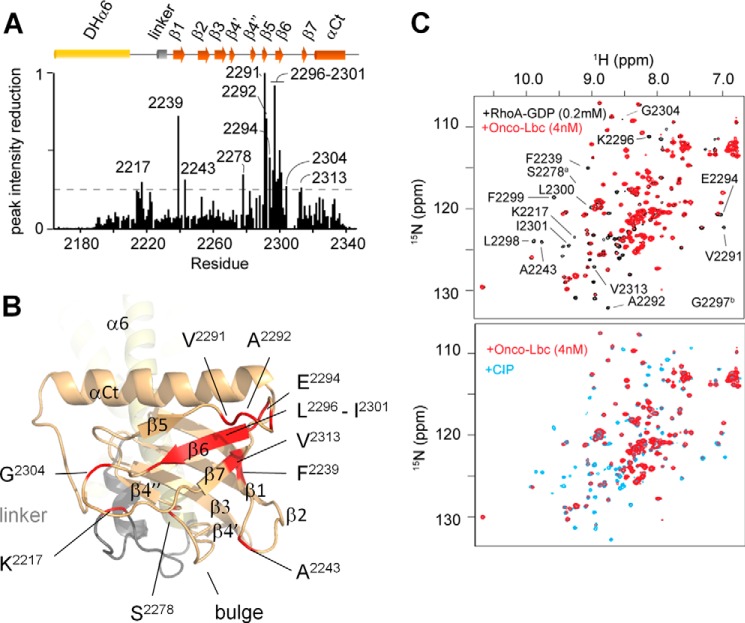
**Mapping of RhoA interaction site.** *A*, binding of RhoA-GTP specifically broadens amide signals in the PH domain following the addition of 4 nm onco-Lbc with peak intensity reductions measured from a ^1^H,^15^N-resolved two-dimensional experiment after 20 min. The *y axis* represents the normalized peak intensity reduction (1 = 100% reduction). *B*, the residues exhibiting line broadening upon RhoA-GTP binding are labeled and map to the exposed β sheet and proximal loops of the PH domain. *C*, the ^15^N-resolved two-dimensional NMR spectra of the AKAP DHαPH domain sample containing RhoA-GDP (1:2 ratio) and GTP (1 mm) are overlaid in the *upper panel* before (*black*) and after addition of onco-Lbc (4 nm) (*red*). The *lower panel* shows the recovery of amide resonances from ^15^N-labeled AKAP DHαPH after addition of calf intestinal alkaline phosphatase (CIP) (*blue*). Signals significantly broadened after addition of onco-Lbc are labeled by the residue. The S2278a and G2297b peaks are weak and located just outside the spectral region displayed, respectively.

The specific association of the Lbc PH domain with activated RhoA was demonstrated by NMR using the ^15^N-labeled DHαPH domain. No perturbations of any DHαPH cross-peak intensities or chemical shifts were observed after sequential addition of GTP and RhoA-GDP (1:2 ratio of DHαPH:RhoA) after more than 20 min, inferring that no binding occurred. However, subsequent addition of 4 nm onco-Lbc immediately yielded a rapid decrease of cross-peak intensities of resonances across the onco-Lbc PH domain, suggesting complex formation with RhoA-GTP in solution due to GEF activity. As the GEF reaction progressed, the intensity of the cross-peaks of the residues Lys^2217^, Phe^2239^, Ala^2243^, Ser^2278^, Val^2291^, Ala^2292^, Glu^2294^, Leu^2298^–Ile^2301^, Gly^2304^, and Val^2313^ was significantly reduced (Fig. 6). These changes circumscribe a surface that has intrinsic protein interaction propensity based on PIER-based protein interaction site prediction (28) and that is centered on the β6 strand. This defines a broad RhoA-GTP-selective docking platform. A second set of cross-peaks corresponding to the bound state could not be observed despite using saturating concentrations of RhoA. This may be due to the high molecular weight of the tight complex formed by DHαPH and RhoA and an intermediate exchange rate on the NMR time scale. This would be consistent with the affinity of ARHGEF11-PH for RhoA-GTPγS in the μm-mm range (42). The slow recovery of most cross-peaks after addition of calf intestinal alkaline phosphatase to the solution confirmed that changes observed were not due to aggregation but instead to a reversible process (Fig. 6*C*). The measurement of progressive resonance intensity changes enabled us to map the docking site of onco-Lbc in a time-resolved manner. The sequence of spectra reproduced the cycle of association and dissociation of the activated RhoA by the PH domain of onco-Lbc and thus demonstrated the specificity of the β6-centered site of the PH domain for the product of the reaction, RhoA-GTP.

##### Mutational Analysis of Lbc Interactions

**FIGURE 7.**
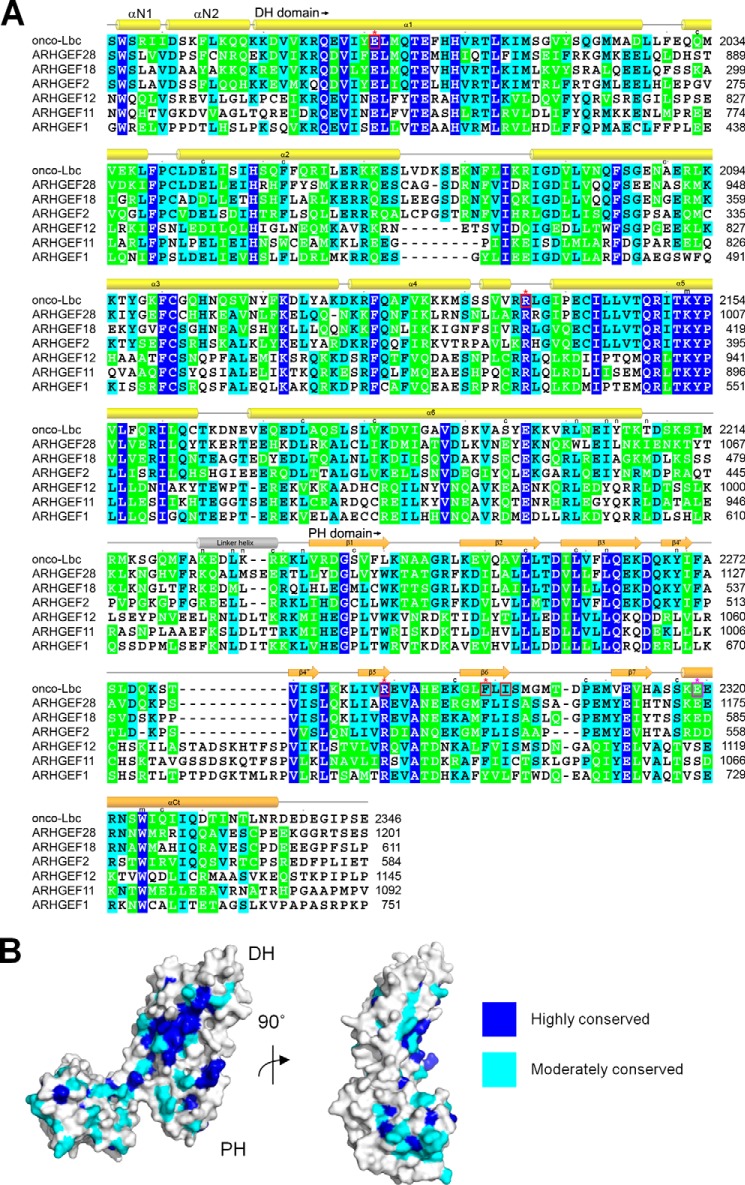
**Structure-based sequence alignment of the ARHGEF family members.** *A*, the amino acid sequences of the tandem DH-PH domains of AKAP-Lbc and its relatives ARHGEF28, ARHGEF18, ARHGEF2, ARHGEF12, ARHGEF11, and ARHGEF1 were aligned by ClustalW and colored by BOXSHADE using Clustal 1.60 values. Absolutely conserved, identical, and similar residues are shaded in *blue*, *aqua*, and *green*, respectively. The residues that, when mutated, reduce or increase GEF activity are *boxed* in *red* and *magenta*, respectively, and indicated with a similarly colored *asterisk*. An “*n*” is placed above those residues that exhibit NMR-based restraints between the DHα6 and linker helices and the PH domain. An “*m*” is placed above those residues in which mutations alter AKAP-Lbc biochemical function including Tyr^2153^ and Trp^2324^. A “*c*” is above those residues that incur substitutions due to missense mutations identified in the Catalogue of Somatic Mutations in Cancer (COSMIC) database (55) including the following: Q2033H, E2044G, F2052L, A2090T, L2174I, V2181L, S2194R, R2229Q, R2229L, S2237N, L2254I, L2259V, K2296R, P2308L, S2317F, and Q2326K. The positions of AKAP-Lbc helices and strands are displayed above the alignment. *B*, surface mapping of the DH-PH tandem according to conservation scores as calculated from the Blosum62 matrix. Highly and moderately conserved residues are represented in *blue* and *cyan*, respectively, and indicate conservation of the functional sites.

Based on the onco-Lbc structural model and similarity with other Lbc RhoGEFs (Fig. 7), mutations were designed to engineer in altered GEF activities. Crystal structures of ARHGEF11 in complex with RhoA as a dimer (Protein Data Bank code 3KZ1) or a monomer (Protein Data Bank code 3T06) were used as a template for manipulating the RhoA interactions (Fig. 5*A*). To test the involvement of the canonical RhoA-GDP binding site, two DH mutations of absolutely conserved residues were generated. The E2001A substitution in the α1 helix reduced the GEF activity to 8.4% (Fig. 5, *B* and *C*), underscoring its significant role in the nucleotide exchange of RhoA. A short sequence in regulatory N-terminal helices αN1 and αN2 that precede the DH domain displays high similarity with other RhoGEF members (Fig. 7). This element is reported to interact with switch 1 of RhoA (39). More precisely, by analogy with ARHGEF11 and ARHGEF12, the Glu^2001^ residue is predicted to stabilize the regulatory elements αN1 and αN2 near the RhoA binding site and could interact with Tyr^34^ of RhoA (39). Mutation of this residue also causes deficient nucleotide exchange in LARG (39). A second mutation in the RhoA-GDP binding site, R2136G in the α4-α5 loop, reduced the GEF activity to 7.4% (Fig. 5, *B* and *C*). The Arg^2136^ residue of onco-Lbc is required for specific recognition of RhoA-GDP residues Asp^45^ and Glu^54^ (17), again confirming this site.

Specific mutations of the Lbc PH domain were designed to test the proposed RhoA-GTP interaction site within the exposed hydrophobic patch centered on β6 and delimited by charged residues. This patch has been proposed as a putative docking site in RhoGEF for proteins including activated RhoA (21, 42). Several mutations were designed to test the RhoA-GTP docking site based on the NMR data, the ARHGEF11 structure (42), and conservation across the RhoGEF family. A pair of hydrogen bonds identified in ARHGEF11 links the residue corresponding to Arg^2289^ of Lbc and Glu^40^ of RhoA. The R2289A mutation reduced the onco-Lbc GEF activity to 10.9% (Fig. 5, *B* and *C*), supporting its important role. Residue Phe^2299^ was found to complement a hydrophobic patch with residues Trp^58^ (21), Phe^59^, and Leu^72^ of RhoA. The F2299A mutation reduced the enzymatic activity to 23.3% of the wild-type form (Fig. 5*C*). Thus, these mutations indicated that docking of the PH domain to Rho-GTP has a vital role in promoting nucleotide exchange.

**FIGURE 8.**
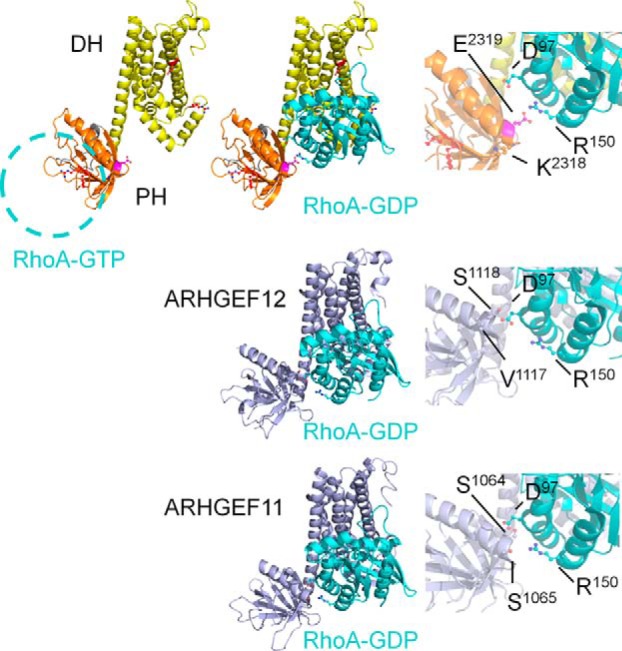
**Putative RhoA binding sites.** The putative location of RhoA-GTP bound to the onco-Lbc PH domain β5-β7 sheet is indicated by a *blue dotted line circle*. Mutated residues are represented by *sticks* and *balls* color-coded according to Fig. 4. The position of RhoA-GDP on the DH domain is inferred from ARHGEF11 and -12. Residues corresponding to Lys^2318^ and Glu^2319^ are represented at the αCt helix of the PH domain for the model of onco-Lbc, ARHGEF11, and ARHGEF12. Residues Asp^97^ and Arg^150^ from RhoA and facing the PH domain are shown in the *enlarged views*.

The αCt helix of Lbc-type PH domains can play a role in stabilizing a RhoA molecule that is bound to the active site of the DH domain (Fig. 8). This is illustrated by ARHGEF11 Ser^1065^ and ARHGEF12 Ser^1118^ residues that interact with RhoA Glu^97^ (38, 39). However, this could infer that the corresponding αCt helix residue in onco-Lbc could generate a repulsive effect on RhoA-GDP interactions. Indeed, an E2319A substitution here yielded enhanced GEF activity close to that of the isolated DH domain, suggesting that this PH domain contact can autoinhibit the GEF activity of onco-Lbc. This negatively charged position is conserved in ARHGEF2, ARHGEF18, and ARHGEF28 (Fig. 7), which hence may share a similar repulsive effect that functionally distinguishes them from the subfamily composed of ARHGEF1, -11, and -12.

## DISCUSSION

Onco-Lbc catalyzes the exchange of GDP to GTP for RhoA in a multistep reaction as revealed by several structural and mutational studies. The mechanism of nucleotide exchange begins with the formation of low affinity complexes by Lbc RhoGEFs engaging RhoA-GDP. The complex formation is directly mediated by the DH domain and is influenced by the αCt helix of the PH domain. This modulation has divergent effects. In ARHGEF11, the PH domain utilizes Met^963^, Glu^969^, and Ser^1065^ in αCt for weak contacts with RhoA as evidenced by chemical shift perturbations (43). These residues correspond to onco-Lbc Leu^2232^, Asp^2235^, and Glu^2319^, respectively. The Glu^2319^ side chain, if similarly involved in RhoA interaction, would coincide with charge clashes based on the ARHGEF11 or ARHGEF12 complexes. Accordingly, GEF activity was boosted when this onco-Lbc residue was replaced with an alanine. The role of charged residues at this position is consistent with mutations carried out in ARHGEF11 where replacement of Ser^1065^ or Asn^1068^ with alanine does not affect RhoA nucleotide exchange kinetics (44). Furthermore, in ARHGEF12, the αCt mutation S1118D demonstrated this positional role in the PH-RhoA interaction (39). Our results suggest that this position in onco-Lbc exhibits a distinctly negative influence over RhoA-GDP binding that differs from ARHGEF11 (42) and ARHGEF12 (39). Our data indicate that the homologous Glu^2319^ residue disfavors the catalytic activation of onco-Lbc. Specifically both the isolated DH domain and the E2319A mutant displayed enhanced activity compared with onco-Lbc.

Mechanistically our results imply that the PH domain of onco-Lbc may undergo a rotation to fully expose the active site of the DH domain. Possibilities of a regulatory influence by the PH domain or lipid binding were discarded as addition of PH domain or lipids failed to modify the GEF activity (data not shown). Possible mechanisms for full activation include an allosteric switch comparable with p63RhoGEF by Gα_q_ whereupon binding to a G-protein the PH domain would undergo a rotation about the linker (45). In fact, AKAP-Lbc was shown to be a downstream effector of the G-protein subunit α_12_ (Gα_12_) that is relayed to RhoA (4, 46). We note that this represents another established difference between ARHGEF1, -11, and -12. The latter all contain a regulator of G protein signaling homology domain distal from the DH-PH and are subject to regulation by Gα_12_ and Gα_13_ (47). In contrast, the ARHGEF-2, -18, -28, and onco-Lbc proteins do not possess such a domain. Hence we infer that this position is a specificity determinant, playing a particularly critical differentiating role in AKAP13 isoforms and exerting more control over RhoA. We also note that the conserved PH-RhoA interface, which includes Glu^2319^ and Asn^2322^, appears to overlap that proposed for inhibitor of NF-κB kinase subunit β (48).

**FIGURE 9.**
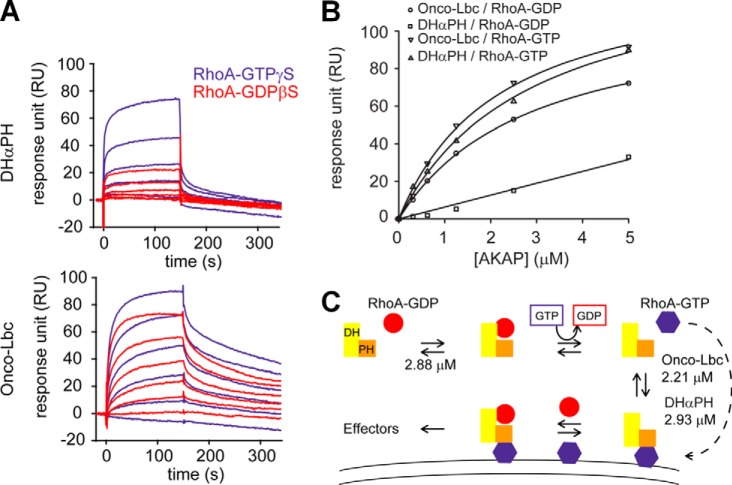
**Binding affinities of RhoA states for onco-Lbc and DHαPH.** *A*, the dissociation constants of the RhoA-GDP·DHαPH, RhoA-GTP·DHαPH, RhoA-GDP·onco-Lbc, and RhoA-GTP·onco-Lbc complexes were determined by surface plasmon resonance as illustrated by Biacore sensorgrams measured for onco-Lbc and DHαPH at varying concentrations (0–5 μm). *B*, the specific association of RhoA-GTP with the PH domain of onco-Lbc (*K_d_* = 2.93 ± 0.37 μm) was contrasted with the inactive GDP-bound RhoA by surface plasmon resonance (*K_d_* > 50 μm). The apparent dissociation constant of onco-Lbc that results from the binding of RhoA at two distinct sites was slightly lower for the active (*K_d_* = 2.21 ± 0.26 μm) *versus* the inactive form of RhoA (*K_d_* = 2. 88 ± 0.11 μm). *C*, model of the feedback mechanism triggered by RhoA-GTP binding. Following the association with RhoA-GDP, the DH domain of onco-Lbc exchanges the nucleotide of RhoA. Once released from the PH domain, RhoA-GTP translocates to membranes by virtue of its farnesylfarnesyl moiety (*dotted arrow*) and specifically recognizes the PH domain of onco-Lbc. The binding of RhoA-GTP by the PH domain does not compete with the GEF activity of the DH domain but rather constitutes a possible mechanism of regulation by orientation of onco-Lbc on the membrane by a PH domain that does not itself contain membrane-interacting sites.

The PH domain of onco-Lbc was found by surface plasmon resonance to associate tightly with the product of the reaction (Fig. 9, *A–C*). The specific interaction of RhoA-GTP was further demonstrated by NMR and mutational analysis. These results are consistent with previous studies showing that mutations within the hydrophobic patch of the PH domain (F2299A and I2301E) reduce the association with RhoA-GTP (21). The dramatic reduction of the GEF activity observed for mutations within this exposed hydrophobic PH patch correlates with the decrease of RhoA-GTP binding. However, the detailed mechanism needs further investigation to resolve how RhoA-GTP association enhances the GEF activity. Conceivably the PH domain could be involved in clearing product from the active site or by transiently forming a multimeric complex such as suggested by the ARHGEF11 crystal structure (42).

**FIGURE 10.**
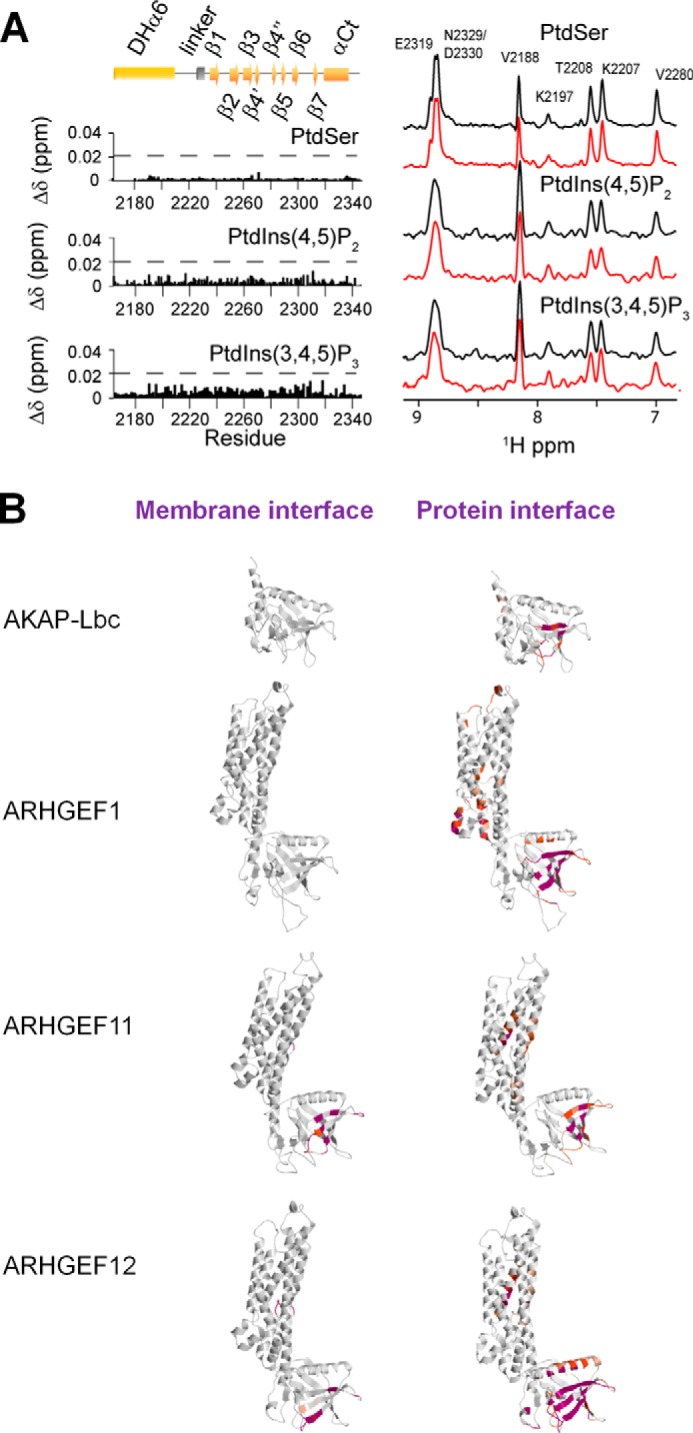
**Assessment of the lipid binding by the PH domain of onco-Lbc.** *A*, chemical shift perturbations were monitored in the ^15^N-labeled AKAP DHαPH domain after addition of dihexanoyl phosphatidylserine (*PtdSer*) (5 mm), PtdIns(4,5)P_2_ (1 mm), or PtdIns(3,4,5)P_3_ (0.57 mm). The absence of specific interaction was shown by the lack of any significant of chemical shift perturbations after each addition. The *dotted line* indicates significant chemical shift perturbations for a positive control protein (FAPP1-PH). Cross-sections of selected amide proton peaks extracted from the heteronuclear single quantum coherence spectra are compared for samples at the start (*black*) and end of the titration (*red*). The peaks are labeled with the corresponding residue. The chemical shift perturbations (Δδ) were calculated as follows: Δδ = [(Δδ_H_)^2^ + (0.15 Δδ_N_)^2^]^1/2^ where Δδ_H_ and Δδ_N_ are the differences of chemical shift in ppm between the start and the end of the titration for the amide proton and nitrogen resonances, respectively. *B*, prediction of membrane interaction sites using MODA and PIER software packages (28, 29). The NMR structure of the DHαPH solution structure and crystal structures of ARHGEF-1, -11, and -12 were used as inputs for predictions. The residues with high (*purple*) and medium (*orange*) propensities for membrane or protein interaction as predicted by MODA and PIER, respectively, are shown as follows: for onco-Lbc, PIER: 2287, 2299, 2302, 2303, 2308, 2310 (*purple*), 2277, 2278, 2286, 2288, 2306, 2307, 2309, 2312 (*orange*); MODA: none; for ARHGEF1, PIER: 445, 448, 449, 451, 539, 658, 704, 713–716, 726, 728, 736, 737, 739 (*purple*), 47, 66, 401, 403, 406, 431, 434, 441, 444, 447, 450, 482, 486, 514, 535, 538, 542, 543, 659, 691, 692, 710, 712, 717–720, 724, 730, 734, 735, 752, 756 (*orange*); MODA: none; for ARHGEF11, PIER: 749, 881, 1046, 1047, 1044, 1055 (*purple*), 743–745, 747, 748, 751, 752, 755, 877, 880, 884, 888, 927, 975, 1021, 1022, 1031–1037, 1048, 1049, 1052–1055, 1058 (*orange*); MODA: 1032, 1034, 1037–1038, 1046, 1048–1051, 1054, 1056 (*red*), 1047, 1052 (*orange*); for ARHGEF12, PIER: 793, 794, 797, 798, 801, 805, 808, 998, 1029, 1059, 1078, 1084, 1091, 1092, 1095, 1102, 1103, 1105, 1120–1122, 1125, 1128, 1129, 1131 (*purple*), 802, 936, 999, 1007, 1010, 1028, 1030, 1060, 1061, 1075–1077, 1080, 1085–1090, 1098, 1101, 1107–1111, 1124 (*orange*); MODA: 868, 918–920, 922–924, 1106–1108, 1088 (*purple*), 921, 1108 (*orange*). The proteins are predicted to associate with membrane-bound RhoA-GTP via the *right-hand* surfaces of their depicted PH domain orientations.

Within cells, onco-Lbc colocalizes along actin stress fibers (49), whereas the isolated PH domain of AKAP-Lbc translocates from the cytosol to the plasma membrane upon stimulation with platelet-derived growth factor (18). The latter translocation depends on phosphoinositide 3-kinase (PI3K) activity, suggesting a phosphoinositide binding function. However, this translocation could also be indirectly caused by polymerization of peripheral actin due to PtdIns(3,4,5)P_3_ production. Moreover, no lipid binding specificity is apparent within the isolated PH domains of AKAP-Lbc or its relatives ARHGEF2, ARHGEF18, and ARHGEF28, although that of ARHGEF3 does exhibits a discernible preference for PtdIns(3,4,5)P_3_ *in vitro*. Similarly, the PH domain of ARHGEF12 does not appear to bind phospholipids in PIP strip assays (50), and the PH domain of ARHGEF1 lacks phosphoinositide binding (51, 52). Because of limitations of these assays, which use lipids adsorbed to nitrocellulose rather than embedded in membrane-like environments, we chose to investigate the interactions using NMR titration and activity assays in liposomes. We found that the AKAP-Lbc PH domain was not affected by liposomes and did not bind directly to PtdIns(4,5)P_2_, PtdIns(3,4,5)P_3_, or phosphatidylserine with any significant affinity despite their presence in the membranes to which it localizes (Fig. 10*A*). Moreover, we note that no member of this ARHGEF family contains a canonical phosphoinositide binding motif in their PH domain (53). Finally, the MODA software, which predicts novel membrane docking surfaces, does not identify any likely membrane binding site on the relevant PH or DH-PH structures (Fig. 10*B*). Together these findings indicate that the ARHGEF proteins including AKAP-Lbc do not directly bind membranes through their PH domains. This does not rule out long range electrostatic complementarity that could orient the rigid DH-PH tandem near a membrane to pick up a RhoA molecule, consistent with PDZRhoGEF studies (43). Indeed the electrostatic surface potentials of the onco-Lbc structures and those of related RhoGEFs suggest that an appropriate electropositive patch is conserved next to the RhoA docking site. Unlike full-length AKAP13, which may localize to membranes via its C1 domain, we propose that onco-Lbc remains soluble as its PH domain does not directly interact with membranes. Instead the DH domain dominates the long range electrostatic membrane attraction alongside its protein interactions complemented by bilayer insertion of the C-terminal prenylated C*AAX* box of RhoA. A previous study (21) has shown that only the membrane-associated activated RhoA can induce a positive feedback effect of ARHGEF11. Thus, further studies using the membrane-bound RhoA are needed to resolve the role of membranes in regulating the catalytic activity of onco-Lbc.

Protein phosphorylation does not appear to play a direct role here in that no appropriate sites on the DH-PH tandem of Lbc are apparent. Instead mitotic cell cycle-dependent phosphorylation of Thr^2398^ and Ser^2400^ is detectable by mass spectrometric analysis of HeLa cell extracts (54) and is found in an unstructured region following the C-terminal helix of the PH domain.

Cancer-linked mutations have been identified that would be predicted to alter Lbc function (55) as shown in Fig. 7. The elucidation of functional sites here provides a basis for future studies of the specific pathological effects and precise mechanisms of action of such cancer-linked mutations. The insights will aid in the structure-based design of targeted therapeutic agents and allow future investigations into the intriguing roles of allostery and membrane association.

## Footnotes

2 The abbreviations used are:

AKAP: A-kinase anchoring protein
GEF: guanine nucleotide exchange factor
PH: pleckstrin homology
DH: dbl homology
LARG: Leukemia-associated RhoGEF
Lbc: lymphoid blast crisis
SAXS: small angle x-ray scattering
MODA: Membrane Optimum Docking Area
Mant-GTP: *2′-/3′-O-(N′-methylanthraniloyl)guanosine 5′-O-triphosphate*
Mant-GDP: *2′-/3′-O-(N′-methylanthraniloyl)guanosine 5′-O-diphosphate*
GMP-PNP: guanosine 5′-(β,γ-imido)triphosphate
GDPβS: guanosine 5′-*O*-(thiodiphosphate)
GTPγS: guanosine 5′-*O*-(thiotriphosphate)
PtdIns(4,5)P_2_: *phosphatidylinositol 4,5-bisphosphate*
PtdIns(3,4,5)P_3_: *phosphatidylinositol* 3,4,5*-trisphosphate*.
